# Association between fatigue and cytokine profiles in patients with ischemic stroke

**DOI:** 10.3389/fneur.2022.1075383

**Published:** 2023-01-23

**Authors:** Inge Kirchberger, Christa Meisinger, Dennis Freuer, Vincenza Leone, Michael Ertl, Philipp Zickler, Markus Naumann, Jakob Linseisen

**Affiliations:** ^1^Epidemiology, Faculty of Medicine, University of Augsburg, Augsburg, Germany; ^2^Institute for Medical Information Processing, Biometry and Epidemiology - IBE, LMU Munich, Munich, Germany; ^3^Department of Neurology and Clinical Neurophysiology, University Hospital Augsburg, Augsburg, Germany

**Keywords:** stroke, fatigue, cytokine, inflammation, biomarkers

## Abstract

**Background:**

Chronic fatigue is a common symptom after a stroke. Studies suggested that chronic fatigue is caused by inflammatory or immunological processes but data are limited and contradictory. Thus, the present study aimed to identify specific biomarkers associated with fatigue in post-stroke patients and replicated the findings in a population-based study.

**Methods:**

We investigated associations between 39 circulating biomarkers of inflammation and fatigue in 327 patients after an ischemic stroke included in the Stroke Cohort Augsburg (SCHANA) study and the “Metabolism, Nutrition and Immune System in Augsburg” (MEIA) study (*n* = 140). The Fatigue Assessment Scale (FAS) was used to assess the severity of fatigue. The serum concentrations of the biomarkers were measured using the Bio-Plex Pro™ Human Cytokine Screening Panel (Bio-Rad, USA). Multiple linear regression models adjusted for possible confounders were used to examine associations.

**Results:**

In patients with stroke, SCGFb was inversely associated [−1.67, 95% confidence interval (CI) (−3.05; −0.29) *p* = 0.018], and in healthy subjects, G-CSF was positively associated [1.56, 95% CI (0.26; 2.87), *p* = 0.020] with an increasing FAS-score, while SCF was positively related in both samples [1.84, 95% CI (0.27; 3.42), *p* = 0.022 and 1.40, 95% CI (0.29; 2.52), *p* = 0.015]. However, after correction for multiple testing, all of these associations lost statistical significance.

**Conclusion:**

The present findings suggested an association between the growth factor SCF and fatigue. Future research on cytokines as possible markers of fatigue should focus on a longitudinal design including a sufficiently large number of study participants to enable testing associations between certain cytokines and sub-groups of chronic fatigue.

## Introduction

There is evidence that fatigue is a common and debilitating symptom after a stroke. Large studies reported that fatigue is present in about 29%−68% of stroke survivors within the first 2 years after the acute event (1–3). A recent study by our group found a fatigue frequency of about 30% in patients with mild impairment 3 and 12 months after the acute stroke event (4). Fatigue is a condition causing distress and loss of functioning due to a lack of energy. It has a negative impact on the quality of life and independence and also on the morbidity and mortality of patients with stroke (5, 6). In the last decade, researchers drew attention to post-stroke fatigue to estimate its prevalence and to examine determinants of its variability (7). The biological mechanisms that cause fatigue are largely unknown or research results are contradictory and incomplete. In previous studies, chronic subclinical inflammation, activation of the immune system, autonomic dysfunction, impairment of the hypothalamic–pituitary–adrenal axis, and neuroendocrine dysregulation have been discussed as biological causes (8). To date, no diagnostic biomarker for fatigue has been identified, this diagnosis still relies on a specific constellation of symptoms (9). Some studies showed associations between cytokine changes in the blood and fatigue. For example, an increase in pro-inflammatory cytokines such as IL-1ß has been associated with fatigue in several chronic diseases (10). However, the data are still insufficient and the results are not consistent, as they depend on the diseases investigated or, in most cases, only a few different biomarkers were determined in small patient samples (9).

In the present study, we, therefore, aimed to identify specific biomarkers associated with fatigue in post-stroke patients in comparison to patients without fatigue using data from the German Stroke Cohort Augsburg (SCHANA) study and replicated the findings in a population-based study. We explored the association between fatigue and a broad range of biomarkers including biologically relevant adaptive immunity cytokines, pro-inflammatory cytokines, and anti-inflammatory cytokines.

## Materials and methods

### Study samples

The present study used data from the “Stroke Cohort Augsburg” (SCHANA) study and the “Metabolism, Nutrition and Immune System in Augsburg” (MEIA) study. The SCHANA study is an observational single-center cohort study that included 945 patients aged 18 years and older with a confirmed ischemic or hemorrhagic stroke who were treated at the University Hospital Augsburg between September 2018 and November 2019 (11). Of the 945 patients, 446 had an ischemic stroke and agreed to blood sample collection and a follow-up survey. Of these, 119 did not complete the 3-month follow-up survey including the questionnaire on fatigue symptoms, leaving 327 for the present analysis. The ongoing MEIA study is a cross-sectional sub-study of the Bavarian Nutrition Survey (BVS-III) on 500 randomly selected participants representative of the adult population aged 18–75 years living in the region of Augsburg, Germany. From the 149 individuals included in the study so far, 140 had completed the questionnaire on fatigue symptoms and were included in the present analysis.

Both studies were performed in accordance with the Declaration of Helsinki. Ethical approvals were obtained from the Ethics Committee of the Ludwig-Maximilians-Universität München (SCHANA: Reference Number: 18-196, BVS-III: Reference Number: 20-334). Written informed consent was obtained from all participants or legal caregivers.

### Data collection

Patients included in the SCHANA study completed a standardized paper-and-pencil questionnaire after the acute stroke event during their hospital stay. This questionnaire covered demographic information, disease history, risk factors, physical activity, and general health status. Clinical data were extracted from the participant's medical records. Three and 12 months after the stroke event, the study participants received a postal questionnaire for follow-up. Participants from the MEIA study were examined at the study center of the Chair of Epidemiology at the University Hospital Augsburg. They completed a questionnaire on a tablet computer, covering demographic information, health conditions, risk factors, and fatigue, among other characteristics.

In both studies, information on sex, age, education [International Standard Classification of Education (ISCED) categories 1–3, categories 4–5], body mass index, smoking (current smoker, ex-smoker, and never-smoker), alcohol consumption according to the Alcohol Disorders Identification Test (AUDIT) (12), fasting status (yes and no) before blood collection, and prior diagnosis of a depressive disorder (yes and no) was collected. Additionally, in the SCHANA study, the time between hospital admission and blood sample collection was documented. Further clinical variables were collected, namely the Modified Rankin Scale classification (13) and the National Institutes of Health Stroke Scale (NIHSS), (14) at hospital admission, and during stroke treatment (systemic thrombolysis and thrombectomy).

### Fatigue assessment

The Fatigue Assessment Scale (FAS), a reliable and valid self-reporting questionnaire for assessing fatigue symptoms, was used in the present study (15). The FAS consists of 10 items with response options from “never” (1) to “always” (5), resulting in a total score between 10 (minimum score) and 50 (maximum score). Persons scoring below 22 are indicated to have no fatigue, subjects with scores between 22 and 35 have a moderate level of fatigue, and persons with scores above 35 can be classified as having a high level of fatigue (16). The FAS was completed by the participants of the SCHANA study 3 months after hospital discharge in a postal survey. Participants in the MEIA study used a tablet computer to complete the questionnaire during their examination at the study center.

### Assessment of blood parameters

From each study participant, about 70 ml of blood was collected either during the hospital stay (SCHANA) or during the visit to the study center (MEIA). Participants from the MEIA study were instructed to fast for 8 h before the study visit. The blood samples were processed and aliquoted into sample tubes at the laboratory of the University Hospital Augsburg, and frozen at −80°C until they were used for biomarker analyses.

Concentrations of 48 cytokines and chemokines were measured in blood serum using the Bio-Plex Pro™ Human Cytokine Screening Panel (Bio-Rad, USA) according to the manufacturer's specifications. In brief, 50 μl of diluted magnetic beads were placed in the wells and washed two times. Then, the standard samples (dilution 1:4: HCSP & IL31, sCD40L; 1:100 VCAM & ICAM), blank, and controls were pipetted into the respective well and incubated between 30 and 60 min on the shaker at 850 +/− 50 rpm at room temperature depending on the panel (60 min IL31 and sCD40L, 30 min VCAM/ICAM and HCSP). This incubation was followed by a three-fold wash step, the addition of the diluted detection antibody, and an incubation of 30 min. After another wash step, streptavadin-phycoerythrin was added and incubated for 10 min. Finally, the plate was washed three more times and resuspended with 125 μl. All incubation steps were performed at room temperature. Measurements were carried out on a Luminex xMAP technology instrument using Bioplex Manager Software.

Measurements were performed in six batches (SCHANA study) or two batches (MEIA), and the differences between the plates were corrected using median normalization. Values specified as below or above the detectable level were replaced by the lowest or highest detectable level according to the manufacturers' reports if present in <25% of the cases. Cytokines with 25% or more values above or below the detection range were excluded from the statistical analyses. Furthermore, cases with outliers in several cytokines were excluded as well as single measurements with extreme outliers. Finally, 39 and 21 cytokines were kept in the SCHANA study and the MEIA study, respectively, for further analyses.

### Statistical analysis

Patient characteristics as well as inflammation markers of both cohorts were described by the median and interquartile range (IQR) in the case of continuous variables or absolute and relative frequencies for categorical variables. For the comparison of the inflammation markers between individuals with and without fatigue, the FAS score was dichotomized using the cut-off value 22 (score <22 means no fatigue; ≥22 means fatigue). Differences in median values of biomarkers between individuals with and without fatigue were tested using the Wilcoxon Rank Sum test.

Multiple linear regression models were used to assess the association between each biomarker (exposure) and level of fatigue (continuous). Biomarker concentrations were log_2_-transformed and σ-standardized to facilitate comparisons.

Regarding the missing value mechanism, which was assumed to be at random (MAR) in the SCHANA study, inverse probability weighting was applied using the following variables: sex, age, education, alcohol intake, smoking status, body mass index, prior diagnosis of depression, Modified Ranking Scale at admission and discharge, NIHSS at admission and discharge, stroke etiology, systemic thrombolysis, and thrombectomy. Furthermore, the linear regression models, investigating the associations between biomarkers and level of fatigue, were adjusted for fasting before blood collection, the time between the acute event and blood collection, and the position of the sample on the 96-well plate. Regression models from the MEIA study data included sex, age, education, alcohol intake, smoking status, body mass index, the position of the sample on the 96-well plate, and prior diagnosis of depression as potential confounding variables.

The *p*-values were corrected for multiple testing using the Benjamini and Hochberg procedure (False Discovery Rate, FDR). All analyses were performed using R software (version 4.1.2).

## Results

Characteristics of the participants from the SCHANA and the MEIA study are presented in Table 1. The studies differed most regarding the median age (SCHANA: 70 years, MEIA: 50 years) education level (higher levels in the MEIA study), hypertension (SCHANA 80.7%, MEIA 25.7%), coronary heart disease or myocardial infarction (SCHANA 18.9%, MEIA 4.3%), and fasting status (SCHANA: 5.9% in a fasting state, MEIA: 99% in a fasting state). In the SCHANA study, patients with fatigue had significantly more often a prior diagnosis of depression, more severe levels of disability according to the Modified Rankin Scale at hospital admission, and higher NIHSS scores at admission than patients without fatigue. In the MEIA study, fatigue was found significantly more often in women and smokers.

**Table 1:** Characteristics of the “Stroke Cohort Augsburg” (SCHANA) and the “Metabolism, Nutrition and Immune System in Augsburg” (MEIA) study.

***n* (%) or median (IQR)**	**SCHANA**				**MEIA**			
	**Total sample (*n* = 372)**	**Fatigue (*n* = 149)**	**No fatigue (*n* = 178)**	*p*-value^*^	**Total sample (*n* = 140)**	**Fatigue (*n* = 35)**	***N*o fatigue (n = 105)**	*p*-value^*^
Age, years	70.0 (60.0, 78.0)	70.0 (59.0, 78.0)	71.0 (60.0, 78.0)	0.563	50.0 (35.0, 61.0)	54.0 (38.0, 60.0)	49.0 (33.0, 61.0)	0.5
**Sex**								
Male	261 (58.5%)	199 (60.9%)	84 (56.4%)	0.129	65 (46.4%)	11 (31.4%)	54 (51.4%)	0.040
Female	185 (41.5%)	128 (39.1%)	65 (43.6%)		75 (53.6%)	24 (68.6%)	51 (48.6%)	
**Education**								
ISCED 1–3	325 (77.2%)	248 (77.5%)	115 (79.9%)	0.360	59 (42.1%)	15 (42.9%)	44 (41.9%)	0.921
ISCED 4–5	96 (22.8%)	72 (22.5%)	29 (20.1%)		81 (57.9%)	20 (57.1%)	61 (58.1%)	
**Smoking**								
Smoker	58 (17.7%)	27 (18.1%)	31 (17.4%)	0.807	22 (15.7%)	5 (14.3%)	17 (16.2%)	0.011
Ex-smoker	138 (42.2%)	60 (40.3%)	78 (43.8%)		55 (39.3%)	21 (60.0%)	34 (32.4%)	
Never smoker	131 (40.1%)	62 (41.6%)	69 (38.8%)		63 (45.0%)	9 (25.7%)	54 (51.4%)	
Alcohol-score (AUDIT)	2.0 (1.0, 4.0)	2.0 (1.0, 4.0)	2.0 (1.0, 5.0)	0.151	3.0 (1.0, 4.0)	3.00 (1.00, 5.00)	3.00 (1.00, 4.00)	
Body mass index, kg/m^2^	26.9 (24.2, 30.4)	27.6 (24.2, 30.9)	26.5 (24.2, 29.7)	0.134	26.2 (22.9, 28.6)	26.4 (23.6, 28.8)	26.2 (22.9, 28.6)	0.559
Fasting before blood collection	21 (6.5%)	7 (4.7%)	14 (8.0%)	0.234	139 (99.3%)	35 (100.0%)	104 (99.0%)	>0.999
Time between hospital admission and blood collection, days	4.65 (3.33, 5.86)	4.80 (3.53, 6.47)	4.47 (3.11, 5.60)	0.064	–	–	–	–
Prior diagnosis of stroke, yes	82 (25.2%)	45 (30.2%)	37 (21.0%)	0.058	2 (1.4%)	1 (2.9%)	1 (1.0%)	0.439
Prior diagnosis of hypertension, yes	264 (80.7%)	127 (85.2%)	137 (77.0%)	0.059	36 (25.7%)	12 (34.3%)	24 (22.9%)	0.180
Prior diagnosis of coronary artery disease or myocardial infarction, yes	57 (18.9%)	28 (20.3%)	29 (17.8%)	0.059	6 (4.3%)	3 (8.6%)	3 (2.9%)	0.165
Prior diagnosis of depressive disorder, yes	25 (7.8%)	19 (13.2%)	6 (3.4%)	0.001	12 (8.6%)	6 (17.1%)	6 (5.7%)	0.073
Fatigue (FAS-score)	21.0 (16.0, 25.0)	26.0 (24.0, 31.0)	16.0 (14.0, 19.0)	<0.001	19.0 (15.0, 21.2)	23.0 (22.0, 24.5)	17.0 (14.0, 20.0)	<0.001
**Modified Rankin Scale (Hospital admission)**								
No symptoms	63 (19.4%)	19 (12.9%)	44 (24.9%)	0.040				
No significant disability	46 (14.2%)	20 (13.6%)	26 (14.7%)					
Slight disability	79 (24.4%)	35 (23.8%)	44 (24.9%)					
Moderate disability	67 (20.7%)	32 (21.8%)	35 (19.8%)					
Moderate severe disability	56 (17.3%)	33 (22.4%)	23 (13.0%)					
Severe disability	13 (4.0%)	8 (5.4%)	5 (2.8%)					
NIHSS (Hospital admission)	1.0 (0.0, 3.0)	2.0 (1.0, 4.0)	1.0 (0.0, 3.0)	<0.001				
Systemic lysis therapy, yes	61 (18.7%)	30 (20.1%)	31 (17.4%)	0.530				
Thrombectomy, yes	18 (5.5%)	9 (6.0%)	9 (5.1%)	0.698				

AUDIT, Alcohol Disorders Identification Test; FAS, Fatigue Assessment Scale; NIHSS, National Institutes of Health Stroke Scale.

* Test of difference between the groups “Fatigue” vs. “No Fatigue” (Chi-square test, Wilcoxon rank sum test, Fisher's exact test).

In the SCHANA study, three biomarkers significantly differed between patients with and without fatigue, namely HGF (*p*-value: 0.048), IL-8 (*p*-value: 0.018), and MIP-1a (*p*-value: 0.025). All three markers showed increased levels in post-stroke patients with fatigue. None of the three inflammatory markers differed between the two groups in the MEIA study, where only the level of the inflammation marker IP-10 (*p*-value: 0.001) was significantly higher in individuals with fatigue compared to subjects without fatigue (Tables 2, 3).

**Table 2:** Serum levels of cytokines (pg/ml; median, 25% quantile; 75% quantile) in 327 individuals with and without fatigue in the “Stroke Cohort Augsburg” (SCHANA) study.

**Cytokine**	**No fatigue^a^**	**Fatigue^b^**	***p*-value^c^**
	***n*** = **178**	***n*** = **149**	
CTACK	928.42 (769.54; 1,126.85)	915.24 (759.37; 1,164.28)	0.788
Eotaxin	61.58 (43.74; 77.92)	63.15 (47.92; 85.18)	0.336
FGF_basic	90.14 (80.07; 97.84)	90.14 (80.61; 99.19)	0.704
G-CSF	113.55 (91.86; 139.22)	113.55 (96.85; 135.06)	0.439
GM-CSF	3.31 (2.36; 4.15)	3.31 (2.25; 4.49)	0.925
GROa	246.24 (189.54; 281.94)	248.87 (210.16; 293.20)	0.266
HGF	484.33 (390.96; 585.21)	509.93 (418.15; 636.72)	0.048
ICAM-1	220,474.67 (181,156.58; 282,402.19)	226,804.90 (189,877.34; 277,822.66)	0.580
IFN-g	16.57 (14.08; 19.05)	16.57 (15.13; 20.7)	0.113
IFNa	20.19 (18.29; 22.54)	20.19 (17.74; 23.36)	0.985
IL-13	2.62 (2.09; 3.58)	2.62 (2.20; 3.54)	0.413
IL-16	51.65 (39.94; 70.14)	54.65 (41.17; 70.96)	0.125
IL-18	54.73 (44.58; 74.51)	57.37 (45.91; 73.40)	0.528
IL-1b	3.72 (3.29; 4.51)	3.72 (3.39; 4.23)	0.780
IL-1ra	392.85 (328.68; 477.01)	408.77 (328.93; 514.23)	0.123
IL-2Ra	83.06 (73.75; 95.85)	85.57 (74.60; 95.62)	0.419
IL-4	4.01 (3.59; 4.54)	3.93 (3.56; 4.42)	0.546
IL-6	3.45 (2.18; 5.18)	3.45 (2.59; 5.49)	0.100
IL-7	17.67 (14.76; 22.17)	17.67 (14.76; 20.54)	0.579
IL-8	10.17 (8.01; 13.04)	11.26 (9.26; 14.15)	0.018
IL-9	137.00 (120.91; 152.89)	140.21 (125.14; 157.09)	0.123
IP-10	367.23 (293.45; 477.06)	370.37 (298.10; 508.20)	0.744
LIF	97.71 (84.20; 117.70)	97.71 (83.60; 117.78)	0.505
MCP-1 (MCAF)	37.52 (29.08; 48.96)	41.66 (30.08; 57.83)	0.064
MCSF	16.82 (14.03; 20.21)	16.95 (13.39; 21.68)	0.570
MIF	673.65 (525.22; 835.03)	668.15 (554.00; 784.88)	0.987
MIG	306.42 (206.34; 425.18)	311.9 (226.09; 537.83)	0.225
MIP-1a	2.79 (2.29; 3.72)	3.18 (2.48; 4.00)	0.025
MIP-1b	89.94 (77.99; 104.40)	92.04 (80.99; 104.45)	0.561
PDGF-bb	2,468.33 (2,058.76; 3,158.71)	2,487.06 (2,188.47; 3,222.69)	0.456
RANTES	11,282.16 (9,326.56; 13,363.63)	11,520.52 (10,067.17; 13,662.09)	0.372
SCF	65.46 (57.47; 77.24)	67.80 (57.47; 79.34)	0.217
SCGFb	114,636.69 (96,868.73; 135,188.21)	114,063.74 (93,628.93; 129,236.23)	0.380
SDF-1a	655.1 (558.91; 732.542)	657.21 (588.85; 744.25)	0.602
TNF-a	55.81 (50.16; 62.04)	57.42 (50.85; 62.93)	0.194
TNFb	145.67 (124.93; 161.07)	147.14 (131.76; 163.65)	0.202
TRAIL	38.07 (31.51; 45.67)	38.85 (33.27; 47.39)	0.335
VCAM-1	807,489.02 (498,094.86; 1,048,921.69)	748,787.72 (531,355.76; 988,939.42)	0.248
VEGF	124.99 (73.98; 170.82)	129.18 (74.80; 175.18)	0.824

a Fatigue Assessment Scale score < 22.

b Fatigue Assessment Scale score ≥ 22.

c Wilcoxon Rank Sum test.

**Table 3:** Serum levels of cytokines (pg/ml; median, 25% quantile; 75% quantile) in 140 individuals with and without fatigue in the “Metabolism, Nutrition and Immune System in Augsburg” (MEIA) study.

**Cytokine**	**No fatigue^a^**	**Fatigue^b^**	***p*-value^c^**
	***n*** = **105**	***n*** = **35**	
CTACK	766.85 (619.16; 932.47)	737.27 (656.65; 920.05)	0.665
EOTXN	39.74 (30.54; 55.39)	31.73 (26.03; 51.90)	0.249
GCSF	6.54 (4.93; 8.15)	6.54 (6.54; 14.64)	0.342
HGF	240.65 (210.70; 282.96)	255.03 (222.56; 308.51)	0.256
ICAM1	1,192,000.00 (1,036,400.00; 1,426,819.16)	1,212,921.73 (1,070,011.14; 1,443,709.58)	0.473
IL16	48.57 (42.42; 56.01)	47.20 (40.00; 56.02)	0.605
IL18	26.99 (19.46; 38.65)	28.58 (15.07; 39.73)	0.834
IL9	392.33 (365.34; 433.66)	384.52 (360.28; 408.64)	0.297
IP10	238.69 (175.63; 336.67)	345.42 (212.14; 589.96)	0.001
LIF	30.82 (14.54; 40.89)	32.25 (18.72; 40.17)	0.682
MCP1	38.65 (28.75; 53.83)	34.88 (25.03; 50.73)	0.361
MIG	128.36 (95.67; 179.48)	161.53 (102.22; 235.75)	0.153
MIP1B	289.86 (274.15; 308.24)	285.40 (263.16; 297.10)	0.174
PDGFBB	2,551.57 (2,127.04; 3,236.37)	2,307.40 (1,821.08; 3,098.47)	0.150
RANTES	13,369.31 (11,319.65; 16,207.52)	13,333.60 (11,169.30; 15,518.25)	0.704
SCF	63.37 (52.89; 77.21)	65.66 (53.99; 77.22)	0.821
SCGFB	143,719.17 (125,862.68; 164,961.70)	141,523.22 (124,507.82; 173,381.46)	0.734
SDF1A	15,98.24 (1,411.68; 1,843.97)	1,649.98 (1,532.55; 1,963.65)	0.207
TNFB	391.49 (362.80; 415.00)	376.84 (339.47; 415.56)	0.210
VCAM1	5,103,650.00 (4,224,100.00; 6,953,250.00)	4,694,300.00 (3,355,025.00; 6,005,350.00)	0.147
SCD40L	147.35 (100.42; 194.08)	139.02 (113.96; 212.10)	0.469

a Fatigue Assessment Scale score < 22.

b Fatigue Assessment Scale score ≥ 22.

c Wilcoxon Rank Sum test.

### Association of biomarkers with fatigue

**Figure 1:**
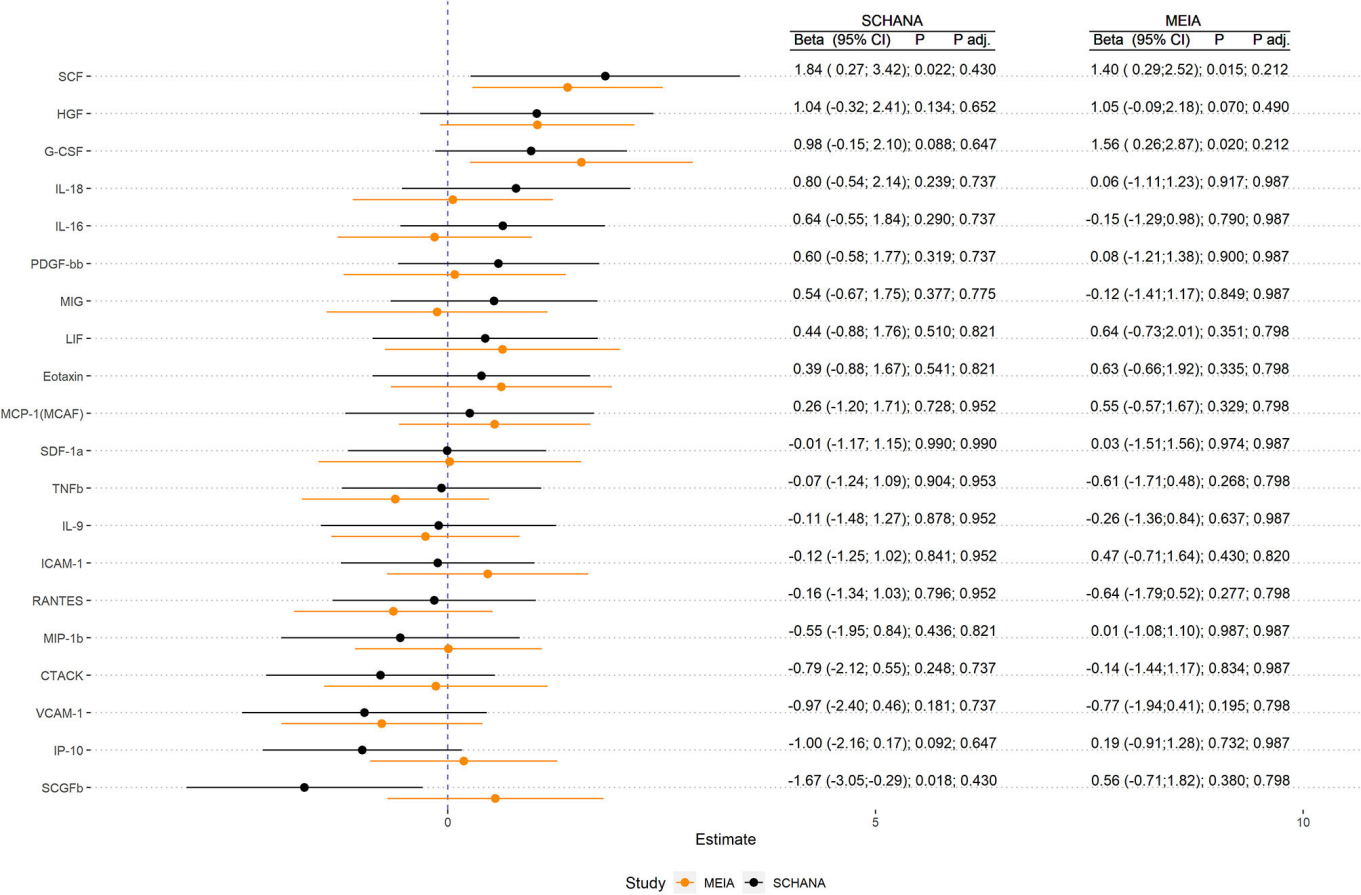
Association of inflammation markers with fatigue in 327 patients with stroke (SCHANA study) and 140 individuals from the general population (MEIA study).

**Figure 2:**
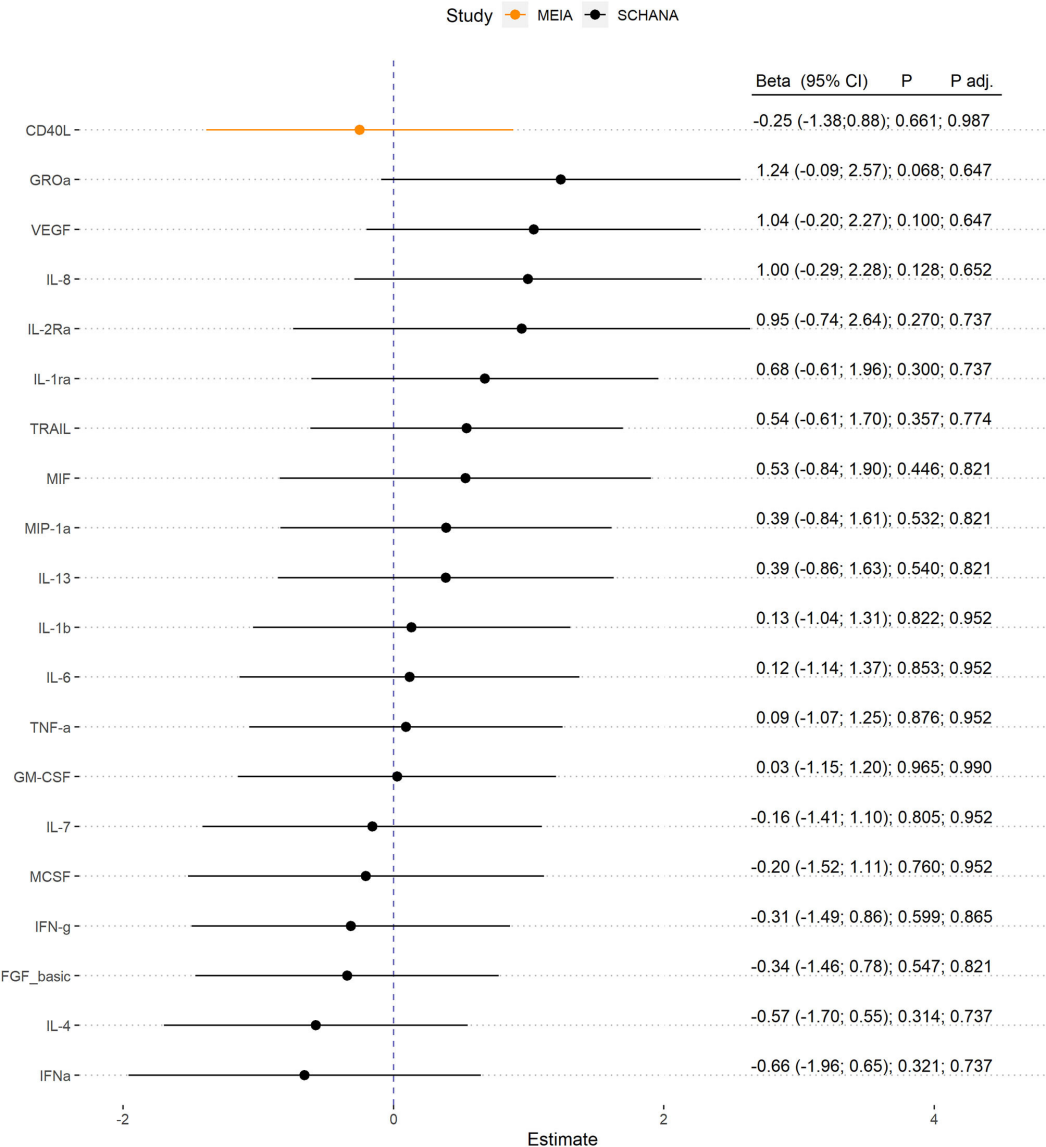
Association of inflammation markers with fatigue. Biomarkers were only evaluable in 327 patients with stroke (SCHANA study) or 140 individuals from the general population (MEIA study).

First, we examined the associations of 39 inflammation markers with fatigue in 327 patients of the SCHANA study. In patients with stroke, two markers were significantly associated with fatigue in multivariable linear regression models, namely SCF [ß = 1.84, 95% confidence interval (CI) 0.27–3.42, *p*-value 0.022], and SCGFb (ß = −1.67, 95% CI −3.05 to −0.29, *p*-value 0.018; Figure 1). In the MEIA study, we could confirm the positive association between SCF and fatigue (ß = 1.40, 95% CI 0.29–2.52, *p* = 0.015) in multivariable analyses, but not the inverse relationship with SCGFb. Contrary to the SCHANA study, we found a significantly positive association of G-CSF with fatigue in the MEIA sample (ß = 1.56, 95% CI 0.26–2.87, *p*-value 0.020). After correction for multiple testing, all of these associations lost significance. Inflammatory markers that could only be evaluated in one of the respective studies also showed no notable associations with FAS scores (Figure 2).

## Discussion

In the present study, we investigated the association between circulating serum levels of 39 inflammatory biomarkers and fatigue in 327 patients after stroke and in 140 subjects from a population-based study. Three inflammatory markers (HGF, IL-8, and MIP-1a) were found to be elevated in patients with fatigue after stroke compared with the group without fatigue. This finding could not be confirmed in the population-based study, where only the marker IP-10 was elevated in subjects with fatigue compared with subjects without fatigue. In patients with stroke, SCGFb was inversely associated and in healthy subjects, G-CSF was positively associated with an increasing FAS score. Furthermore, in both studies, we found an independent positive association between SCF and fatigue in multivariable linear regression analyses. However, the associations lost statistical significance after correction for multiple testing.

In patients with post-stroke fatigue, the markers hepatocyte growth factor (HGF), interleukin-8 (IL-8), and macrophage inflammatory protein-1 alpha (MIP-1a) were elevated compared to patients without fatigue. The proinflammatory cytokine IL-8, also known as CXCL8, is secreted particularly by endothelial cells, monocytes, epithelial cells, and fibroblasts and acts as a chemoattractant and a potent angiogenic factor (17). In contrast to our study, the study by Groven et al. (18) did not detect increased IL-8 levels in patients with fatigue compared with controls. HGF is a pleiotropic cytokine secreted by mesenchymal cells that acts mainly on epithelial and endothelial cells but also on hematopoietic progenitor cells and T cells (19). To date, this marker has not been associated with the presence of fatigue in literature. However, studies have examined HGF in relation to depression, with one study finding significantly lower serum HGF levels in older individuals with major depression compared with controls (20). In another study, HGF levels were significantly elevated in women with postpartum depressive symptoms (21). The chemokine MIP-1a, also called CCL3, is produced by macrophages and monocytes after stimulation with bacterial endotoxin or proinflammatory cytokines such as IL-1ß. It is expressed by all hematopoietic cells and some tissue cells such as fibroblasts, epithelial cells, vascular smooth muscle cells, or platelets upon activation (22). In a study including women with early-stage breast cancer, MIP-1a was found to be associated with fatigue after receipt of chemotherapy but not before (23). In the present population-based sample, but not in post-stroke patients, the proinflammatory cytokine interferon gamma-induced protein (IP-10), also known as CXCL10, was significantly increased in subjects with fatigue compared to the non-fatigue group. This finding is in contrast to a recent study which found no differences regarding IP-10 levels when comparing patients with fatigue vs. healthy controls (18). IP-10 plays a critical role in inflammatory diseases and regulates immune responses through the activation and recruitment of leukocytes including T cells, eosinophils, monocytes, and NK cells (24). It is secreted by a variety of cells including monocytes, neutrophils, endothelial cells, keratinocytes, fibroblasts, mesenchymal cells, dendritic cells, astrocytes, and hepatocytes (24).

In both study samples, in multivariable-adjusted linear regression models, we found a possible association between SCF and fatigue. SCF is a glycoprotein that exists both in a membrane-bound form and as a soluble protein (25). As a cytokine and a member of the hematopoietic growth factors, it is involved in the early stages of hematopoiesis (25). *In vitro* studies found that SCR in combination with other hematopoietic growth factors, such as granulocyte colony-stimulating factor (G-CSF), stimulates hematopoietic progenitor cells (26). *In vivo* studies showed that SCF synergizes with other growth factors and enhances the mobilization of peripheral blood progenitor cells in combination with G-CSF (26). In addition to the role of SCF on the hematopoietic system, studies suggested that it may play a role in the development and function of germ cells (27) and melanocytes (28). Moreover, SCF may also play a role in the central nervous system. It appears that it is overexpressed by neurons after brain injury and mediates the migration of neural stem cells to the site of brain injury (29). Presumably, SCF also plays a role in inflammatory processes (25). It seems that it is upregulated under inflammatory conditions (30) and plays a compensatory role in trying to limit the extent of damage after stroke by favoring reparative mechanisms (31).

A recent study, including 192 patients with chronic fatigue and 392 healthy controls, examined the association between multiple markers of inflammation and chronic fatigue severity (32). A total of 17 cytokines, including SCF, had a statistically significant upward linear trend correlating with fatigue severity, among others with SCF. Since, in our investigation, a potential association between SCF and fatigue could also be observed in the general population, SCF levels in post-stroke patients may be related to fatigue regardless of compensatory mechanisms. However, although SCF was suggestively associated with fatigue in both study samples in our analysis, the results should be interpreted cautiously. Further studies are needed to investigate whether and if so, what role SCF plays in the development and severity of fatigue. Interestingly, there is increasing evidence that neuroinflammation is associated with chronic fatigue (33). Microglia, the innate immune cells of the central nervous system, have pro-inflammatory or neuroprotective properties and hence may play a central role in mediating this neuroinflammation. These neuroglial cells could be a link among inflammation, immunity, stress response, and homeostasis of the central nervous system. The hypothesis that chronic neuroinflammation and central nervous system dysfunction may be involved in disease mechanisms has to be the subject of further research activities (34).

Comparing the findings of different studies on the relationship between inflammatory markers and fatigue is difficult. Cytokine levels in peripheral blood are subject to fluctuations that may be influenced by various factors such as sleep, obesity, smoking, time of day, and method of analysis (35, 36). Furthermore, cytokines are released by immune cells in small amounts and have a broad range of effects, such as pleiotropic, synergic, and antagonistic effects, among others (37, 38). Thus, it may be difficult to detect clinically important concentrations of circulating cytokines, because most of their effects are likely to be local. Finally, cytokines are likely to be one factor in a complex network. Also, differences regarding the measure of fatigue, different study populations, or a lack of statistical power can influence the study results. So far, the pathophysiology of chronic fatigue syndrome is poorly understood. Different comorbidities and underlying diseases could be present in patients with fatigue, who thus represent a heterogeneous group at different stages of their disease (9, 39, 40). Currently, there seems to be no clear picture of which cytokines may play what role regarding chronic fatigue (41). In prior studies, the most frequently studied pro-inflammatory cytokines were IL-1β, TNF, and IL-6. However, the results mainly demonstrated that they are not raised in subjects with chronic fatigue syndrome in comparison to controls (41). In meta-analyses, the cytokine with the best support of involvement in chronic fatigue syndrome was the multifunctional cytokine transforming growth factor beta (TGF-β) as a potential biomarker for categorizing individuals with chronic fatigue into sub-groups (32, 41). However, in prior studies, no association between TGF-β and the severity of fatigue could be found (32).

The present study has several limitations. First, due to the cross-sectional design of the studies, no causal relationships could be investigated. Second, the sample size was relatively low regarding the number of inflammatory markers measured. Thus, after correction for multiple testing, no significant associations remained. Third, blood was sampled mostly in a non-fasting state and at different time points after the acute stroke event. Fourth, the study samples differ regarding the age of the included participants. Fifth, although only patients with ischemic stroke were included in the study, they differ regarding etiology. Strengths of our study include the availability of two independent study samples to investigate the research question. Furthermore, in both samples, the same instrument for the assessment of fatigue was available and the same biomarker profile was measured.

In conclusion, the present findings point toward an association between the growth factor SCF and fatigue. However, there is no clear picture to support the hypothesis that pro-inflammatory circulating cytokines are related to fatigue. Future research on cytokines as possible markers of fatigue should be conducted in a longitudinal design. Also, further studies should include the measurement of cytokines in different body fluids and multiple measurements in a sufficiently large number of study participants, to enable testing associations between certain cytokines and sub-groups of chronic fatigue.

## Data availability statement

The datasets presented in this article are not readily available because of ethical and privacy restrictions. Requests to access the datasets should be directed to: CM, christine.meisinger@med.uni-augsburg.de.

## Ethics statement

The studies involving human participants were reviewed and approved by Ethics Committee of the Ludwig-Maximilians-Universität München, Germany. The patients/participants provided their written informed consent to participate in this study.

## Author contributions

IK: conceptualization, methodology, and writing—original draft. CM: conceptualization, investigation, methodology, and writing—original draft. DF and VL: formal analysis and writing—review and editing. JL and MN: conceptualization, resources, supervision, and writing—review and editing. PZ and ME: conceptualization, investigation, and writing—review and editing. All authors contributed to the article and approved the submitted version.
